# Identification of patients with suspected NSTE-ACS in the observe zone: evaluating GRACE 1.0 score and a biomarker panel for risk stratification and management optimization

**DOI:** 10.1007/s00392-025-02642-3

**Published:** 2025-04-14

**Authors:** Mustafa Yildirim, Christoph Reich, Christian Salbach, Moritz Biener, Matthias Mueller-Hennessen, Nils Arne Sörensen, Paul Michael Haller, Stefan Blankenberg, Johannes Tobias Neumann, Raphael Twerenbold, Norbert Frey, Evangelos Giannitsis

**Affiliations:** 1 Department of Internal Medicine III, Cardiology, University Hospital of Heidelberg, Im Neuenheimer Feld 410, 69120 Heidelberg, Germany; 2 Department of Cardiology, University Heart and Vascular Center Hamburg, University Medical Center Hamburg-Eppendorf, Hamburg, Germany; 3 DZHK (German Centre for Cardiovascular Research), Partner Site Heidelberg/Mannheim, Heidelberg, Germany; 4 DZHK (German Center for Cardiovascular Research), Partner Site North, Hamburg, Germany

**Keywords:** Observe zone, Grey zone, Cardiac troponin, High sensitivity, Copeptin, Acute coronary syndrome, Emergency department

## Abstract

**Background:**

Current guidelines recommend additional diagnostic work-up for patients with suspected non-ST-elevation acute coronary syndrome (NSTE-ACS) triaged in the observe zone using accelerated diagnostic protocols. This study assessed the effectiveness of combining the Global Registry of Acute Coronary Events (GRACE) 1.0 score with additional non-cardio-specific biomarkers for risk stratification in the observe zone.

**Methods:**

A total of 6789 patients with suspected NSTE-ACS were enrolled over 24 months, with 961 (21.8%) assigned to the observe zone. A classification and regression tree (CART) analysis dichotomized risk using the GRACE-score and additional biomarkers beyond high-sensitivity cardiac troponin including C-reactive protein < 10 mg/dL, N-terminal pro-B-type natriuretic peptide < 300 ng/L, D-dimers < 5 mg/L, estimated glomerular filtration rate > 30 mL/min/1.73m^2^, Copeptin < 10 pmol/L, and hemoglobin > 10 g/dL. The primary endpoint was 1-year all-cause mortality, validated using the Biomarkers in Acute Cardiac Care (BACC) cohort.

**Results:**

A low GRACE 1.0 score < 109 points was found in 37.6% of observe zone patients, showing a negative predictive value of 98.6% and sensitivity of 89.8% for death. Adding biomarker information reduced predicted 1-year-mortality from 1.38% with the GRACE-score alone to 0.46% when none of the biomarkers were above cutoff (prevalent in 22.7%). The proportion of protocol-eligible patients increased from 22.7 to 37.6%, with no events within 30 days. Findings were confirmed in the BACC cohort.

**Conclusion:**

A low GRACE 1.0 score combined with ≤ 1 elevated biomarker significantly improves mortality prediction in the observe zone, helping identify low-risk patients for further out-of-hospital diagnostic work-up, potentially decongesting crowded emergency departments.

*Registration* URL: https://www.clinicaltrials.gov; Unique identifier: NCT05774431.

**Graphical abstract:**

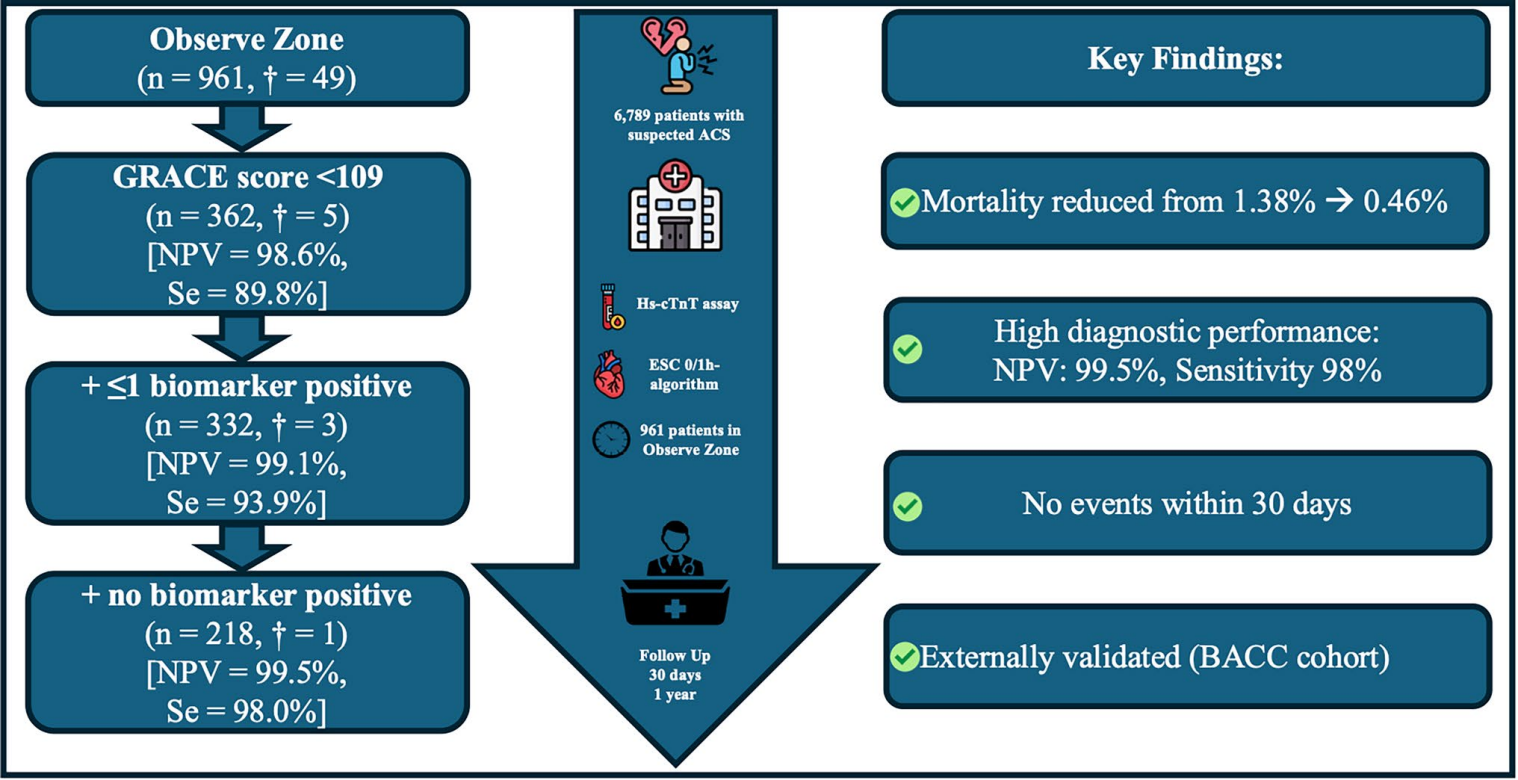

**Supplementary Information:**

The online version contains supplementary material available at 10.1007/s00392-025-02642-3.

## Introduction

As a consequence of the incremental implementation of accelerated triage protocols that use optimized troponin-based cutoffs for rule-out and rule-in, a new triage category called “observe zone” has emerged that is associated with diagnostic uncertainty and adverse prognosis [1–4]. Accordingly, 2020 European Society of Cardiology (ESC) guidelines on non-ST-elevation acute coronary syndrome (NSTE-ACS) [5] started to recommend a third troponin measurement after 3 h to increase the diagnostic yield and to refine risk stratification. Unfortunately, existing protocols either lack appropriate safety or leave up to a third of patients in the observe zone [6]. Notably, the additional measurement of other biomarkers beyond high-sensitivity cardiac troponin (hs-cTn) and natriuretic peptides is discouraged for diagnosis and risk stratification by ESC 2020 guidelines on NSTE-ACS [5]. As an alternative strategy, the 2022 American College of Cardiology (ACC) Expert Consensus on Chest Pain in the emergency department (ED) [7] suggests implementing clinical risk scores to further stratify these patients. However, a recent secondary analysis of the High-Sensitivity Cardiac Troponin I Assays in the United States (HIGH-US) study [8] revealed that risk scores are unlikely to improve triage without additional troponin measures and imaging. Whether artificial intelligence and machine learning (ML) are able to improve risk stratification in the observe zone is illusive, at the moment.

Therefore, and in the absence of established guidance, we tested whether a combination of the established Global Registry of Acute Coronary Events (GRACE) 1.0 score [9] and a low biomarker-related risk could improve risk stratification within the observe zone. The GRACE 1.0 score consists of eight clinical parameters, including age, heart rate, systolic blood pressure, serum creatinine, Killip class, cardiac arrest at admission, ST-segment deviation, and elevated cardiac enzymes. Beyond hs-cTn, hemoglobin, estimated glomerular filtration rate (eGFR) and C-reactive protein (CRP) were measured routinely, and additional biomarkers were requested at the discretion of attending physicians for diagnosis of acute or chronic comorbidities including infection, venous thromboembolism, or heart failure.

## Methods

### Study population

The RAPID-CPU registry is a monocenter observational study that enrolled consecutive patients presenting with suspected NSTE-ACS between July 1st, 2016, and June 30th, 2018, at the ED of Heidelberg University Hospital. A flow diagram for included and excluded patients within this study is shown in supplement Fig. 1. Details on the study population and interventions have been previously published [10]. Briefly, patients were eligible for enrollment if they presented with clinically suspected acute coronary syndrome (ACS), based clinically on a broad spectrum of symptoms including atypical chest pain or dyspnea. Exclusion criteria included missing 0-h or consecutive measurements if serial measurements were required, documented AV nodal re-entry tachycardia (AVNRT), acute heart failure due to known structural heart disease, primary pulmonary disease without suspected ACS, traumatic chest pain with preceding thorax injury, dysfunction or alarm of an implantable cardiac device (ICD), chronic hemodialysis, inadequate command of English/German language, or permanent residence in a foreign country. Patients were not excluded for severe chronic kidney disease, older age, chronic heart failure, suspected acute heart failure due to myocardial infarction (MI), atrial fibrillation, or missing 3-h measurements of the high-sensitivity cardiac troponin T (hs-cTnT) assay, as a third hs-cTn measurement at 3 h after the initial measurement was not obligatory until the 2015 ESC Guidelines [11].

Using the validated ESC 0/1-h protocol, myocardial infarction was ruled out in patients presenting more than 3 h after symptom onset with an initial hs-cTnT below the limit of detection (LoD: < 5 ng/L), or if the initial hs-cTnT was < 12 ng/L with an absolute concentration change < 3 ng/L within the first hour. Patients were classified as 'rule-in' if the initial hs-cTnT concentration was ≥ 52 ng/L or if there was an absolute concentration change ≥ 5 ng/L within the first hour. Patients who did not fulfill either Rule-out or Rule-in criteria were categorized into the observe zone, and only these patients qualified for the present study. Diagnosis of MI was diagnosed by the treating physician using hs-cTnT and at that time of study conduct the criteria of the 2015 ESC guidelines [11], and the 4th version of the Universal Definition of Myocardial Infarction (UDMI) [12]. The GRACE 1.0 risk score was calculated using the Fox model for death between hospital admission and 6 months [9]. It integrates eight clinical parameters: age, heart rate, systolic blood pressure, serum creatinine, Killip classification, cardiac arrest at admission, ST-segment deviation on ECG, and elevated cardiac enzymes, following the original GRACE definitions. Scores of < 109, 109–140, and > 140 points categorize patients into low-, intermediate-, and high-risk groups, respectively.

External validation of the protocol was executed in the Biomarkers in Acute Cardiac Care (BACC) cohort which has been described earlier (Clinical Trials Identifier: NCT02355457) [13]. This observational study is an ongoing, prospective cohort study including patients who presented to the emergency department at the University Hospital of Hamburg with suspected non-ST-elevation acute coronary syndrome (NSTE-ACS).

### Laboratory analyses

Plasma high-sensitivity cardiac troponin T (hs-cTnT) was measured with the Elecsys® Troponin T high-sensitivity assay (Roche Diagnostics) on a Cobas e411 immunoassay analyzer. LoB, LoD, 10% coefficient of variation (CV), and 99th percentile cut-off values were determined to be 3 ng/L, 5 ng/L, 13 ng/L and 14 ng/L [14, 15]. Copeptin in plasma samples at baseline (0 h) was measured with the copeptin proAVP assay on the KRYPTOR compact plus (BRAHMS Thermo Fisher Scientific). Detection limit, precision of 20% CV and 95th cut-off values for the copeptin proAVP assay were found to be 0.69 pmol/L, 1.08 pmol/L, and 9.8 pmol/L [16, 17]. An elevated copeptin was defined at concentrations > 10 pmol/L. NT-pro BNP was measured using the Siemens Atellica® using the general rule-out cutoff of 300 ng/L per ICON trial [24]. All other biomarkers including CRP (< 10 mg/dL), calculated eGFR (> 30 mL/min/1.73 m^2^), as well as hemoglobin (> 10 g/dL) were measured in the central laboratory on automated analyzers (Siemens) at established cutoffs [18–21]. The additional measurement of other biomarkers was not mandatory and was either part of laboratory routine, such as CRP, serum creatinine, eGFR and hemoglobin, or was ordered by the attending physician per clinical need for suspected comorbidities or underlying differential diagnoses of suspected ACS such as N-terminal pro-B-type natriuretic peptide (NT-proBNP) for suspected structural heart disease or acute heart failure, while D-dimer and copeptin were assessed for suspected venous thromboembolism, following the 2019 ESC guideline recommendations for pulmonary embolism [22] GFR was estimated based on serum creatinine using the race-independent CKD-EPI (Chronic Kidney Disease Epidemiology Collaboration) equation [23].

### Follow-up

Patients were followed for a median of 12 months for the occurrence of all-cause death. Follow-up was accomplished using telephone, questionnaire, patient’s hospital notes, the family physician’s records, and the municipal registry on vital status. The study protocol was approved by the Ethics Committee of the Medical Faculty of Heidelberg. The trial was registered at ClinicalTrials.gov. (Clinical Trails.gov Identifier: NCT03111862).

### Outcome and data collection

The primary outcome was 1-year all-cause mortality. This is generally considered the most useful outcome in identifying patients at very low risk of poor outcomes. Rates of cardiovascular (CV) death, MI, stroke, or other outcome events were not collected systematically. Data entry was performed by a dedicated research nurse, physician, or medical student at each site, and data collection included patient characteristics, clinical variables, and laboratory results at presentation required to calculate the GRACE-score. Data for determination of outcome measures were also collected.

### Statistical analysis

Continuous variables were tested for normal distribution and were presented either as means with 95% CIs, or as medians with minimum and maximum. The normality of data distribution was assessed by the Kolmogorov–Smirnov test. Groups were compared using the *χ*^2^ test for categorical variables and Kruskal–Wallis test for continuous variables. Kaplan–Meier curves and the log-rank test were used. A classification and regression tree (CART) analysis was conducted with all-cause death as the primary outcome. The model identified GRACE 1.0 < 109 as the variable most strongly associated with outcomes. The absence of a positive biomarker or the presence of ≤ 1 biomarker served as the splitting points to best classify observations into groups. This combination of factors results in an easily visualized tree-like plot with corresponding event rates. Prevalence of individuals and events is provided for each tree branch. Our CART analysis focused on the model's ability to identify patients at the lowest risk, emphasizing the rule-out part. The model was developed using the entire training set, and the resulting tree structure was validated internally using bootstrapping and externally in the BACC cohort. All hypothesis testing was two-tailed and *p* values < 0.05 were considered statistically significant. All statistical analyses were carried out using the R software (version 4.3.0, R Foundation for Statistical Computing, Vienna, Austria) and MedCalc 20.111 (MedCalc Software bvba, Ostend, Belgium).

## Results

### Baseline characteristics

A total of 6789 patients were enrolled during a period of 24 months. The ESC-0/1 h algorithm was applicable to 4,413 patients due to missing values, because a 3rd hs-cTnT value was not recommended per 2015 ESC Guidelines and hence was not available [11]. Of these, 1146 were classified into the "Rule-in" group, 2306 were classified into the "Rule-out" group, and 961 were classified into the observe zone (derivation cohort). Among the patients in the observe zone, 51.6% were admitted, while 48.4% were discharged. Patients who survived beyond 1 year had a higher discharge rate (49.8%) compared to patients who died within 1 year (22.4%; *p* = 0.0002). Following further diagnostic work-up and reclassification, 7.2% were diagnosed with NSTEMI, and the 1-year mortality rate was 5.1% (49 out of 961). Median follow-up for the cohort was 444 days, with an interquartile range (IQR) of 339–642 days. Baseline parameters of patients within the observe zone spit by survival status are presented in Table 1. Non-survivors were older (*p* < 0.0001), presented with higher median systolic blood pressure (*p* = 0.0001) and a significantly higher median GRACE-score (*p* < 0.0001). They had higher concentrations of cardiac biomarkers such as hs-cTnT and D-dimer (both *p* < 0.05) and lower levels of hemoglobin compared to survivors (*p* < 0.0001). Additionally, non-survivors had a higher prevalence of cardiovascular comorbidities, including coronary artery disease (CAD), MI, and hypercholesterolemia (all *p* < 0.05).

**Table 1:** Baseline characteristics of patients in the observe zone split by survival status, RAPID-CPU registry

	All (*n* = 961)	Non-survivor (*n* = 49)	Survivor (*n* = 912)	*p* value
Age [years], median (IQR)	74 (62–81)	81 (76–89)	73 (61–80)	< 0.0001
Female gender, *n* (%_all_)	367 (38.2)	21 (5.1)	346 (36.0)	< 0.0001
Heart rate [bpm], median (IQR)	76 (66–87)	82 (71–97)	76 (66–87)	0.0204
Systolic pressure [mmHg], median (IQR)	152 (139–169)	148 (129–164.5)	152 (140–169)	< 0.0001
GRACE-score, median (IQR)	121 (95–140)	151 (125–174.5)	118 (95–138)	< 0.0001
Low, * n* (%_all_)	362 (37.7)	5 (0.5)	357 (37.1)	< 0.0001
intermediate, * n* (%_all_)	365 (38.0)	16 (1.7)	348 (36.2)	< 0.0001
high, * n* (%_all_)	234 (24.3)	27 (2.8)	207 (21.5)	< 0.0001
*Symptoms*				
Time since onset < 3 h, * n* (%_all_)	186 (19.4)	10 (1.0)	176 (18.3)	0.295
Chest pain, * n* (%_all_)	554 (57.6)	22 (2.3)	532 (55.4)	0.001
Dyspnea, * n* (%_all_)	193 (20.1)	13 (1.4)	180 (18.7)	0.0120
*Laboratory*				
hs-cTnT 0 h [ng/L], median (IQR)	16 (12–24)	21 (15.8–34.8)	16 (12–23.5)	< 0.0001
hs-cTnT 1 h [ng/L], median (IQR)	15 (10–20)	18.5 (14–26.5)	15 (10–20)	0.0073
NT-proBNP [ng/L], median (IQR)	769.5 (198–3402)	4794 (819.8–7895.3)	688 (181–2883.5)	< 0.0001
GFR [mL/min/1.73 m^2]^], median (IQR)	73 (54.3–87.9)	60 (46.5–83.2)	73.3 (55.2–88.3)	0.0180
D-dimer [ng/L], median (IQR)	0.45 (0.28–0.92)	5.4 (1.7–8.5)	0.44 (0.28–0.87)	0.0002
Copeptin [pmol/L], median (IQR)	7.6 (4.4–15.0)	12.2 (6.1–26.0)	7.5 (4.4–14.6)	0.4343
Hemoglobin [pg/L], median (IQR)	13.4 (12.1–14.6)	12.2 (11.0–13.6)	13.4 (12.4–14.6)	< 0.0001
CRP [mg/dL], median (IQR)	3.4 (1–11.6)	13.7 (3.6–30.7)	3.1 (1–10.5)	< 0.0001
*History*				
CAD, * n* (%_all_)	478 (49.7)	32 (3.3)	446 (46.4)	0.0254
Myocardial infarction, * n* (%_all_)	235 (24.5)	19 (2.0)	216 (22.5)	0.0167
CABG, * n* (%_all_)	108 (11.2)	9 (0.9)	99 (10.3)	0.1057
Congestive heart failure, * n* (%_all_)	266 (27.7)	19 (2.0)	247 (25.7)	0.0749
Smoking current, * n* (%_all_)	139 (14.5)	10 (1.0)	129 (13.4)	0.1832
Art. hypertension, * n* (%_all_)	782 (81.4)	40 (4.2)	742 (77.2)	0.3669
Diabetes mellitus, * n* (%_all_)	284 (29.6)	17 (1.8)	267 (27.8)	0.3595
Dyslipidemia, * n* (%_all_)	525 (54.6)	31 (3.2)	494 (51.4)	0.0433
Family history of CAD, * n* (%_all_)	233 (24.2)	14 (1.5)	219 (22.8)	0.2753
*Therapeutic work-up*				
Coronary angiography, * n* (%_all_)	283 (29.5)	10 (1.0)	273 (28.4)	0.1543
PCI, * n* (%_all_)	127 (13.2)	5 (0.5)	122 (12.7)	0.5231
CABG, * n* (%_all_)	20 (2.1)	0 (0)	20 (2.1)	0.3192

bpm, beats per minute; CABG, coronary artery bypass graft; CAD, coronary artery disease; COPD, chronic obstructive pulmonary disease; CRP, C-reactive protein; GFR, glomerular filtration rate; Hs-cTnT, high-sensitivity cardiac troponin T; NT-proBNP, N-terminal pro-B-type natriuretic peptide; PCI, percutaneous coronary intervention. Percentages may not total 100 because of rounding

**Fig. 1:**
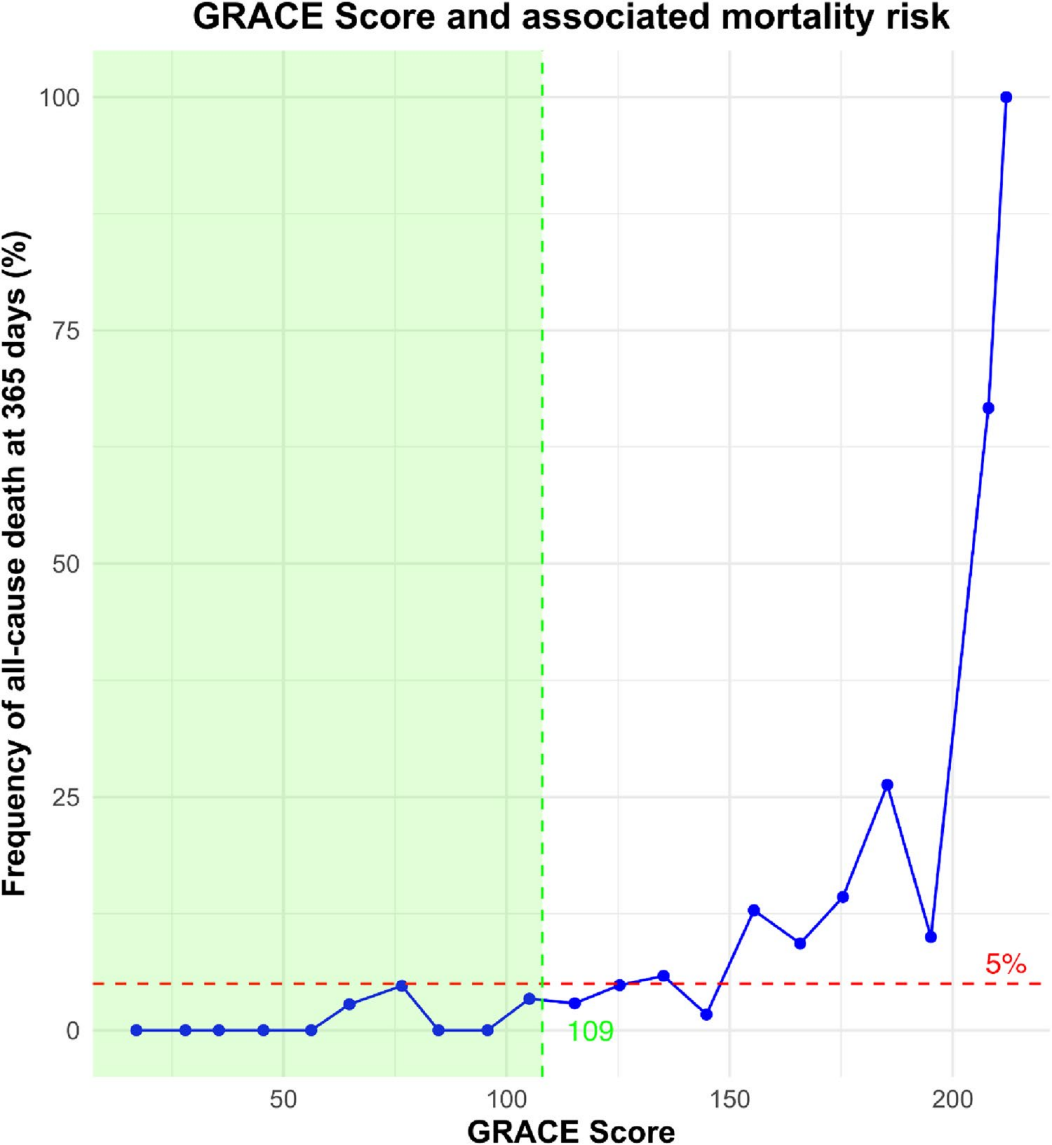
Frequency of all-cause death over 365 days in relation to GRACE-score. The *y*-axis represents the frequency of all-cause death at 365 days, while the *x*-axis shows the GRACE-score. A red line marks a 5% mortality threshold, which coincides with a GRACE-score of 109 points, delineating the low-risk group. The area from 0 to 109 GRACE points is shaded green, indicating the region of low mortality risk

**Fig. 2:**
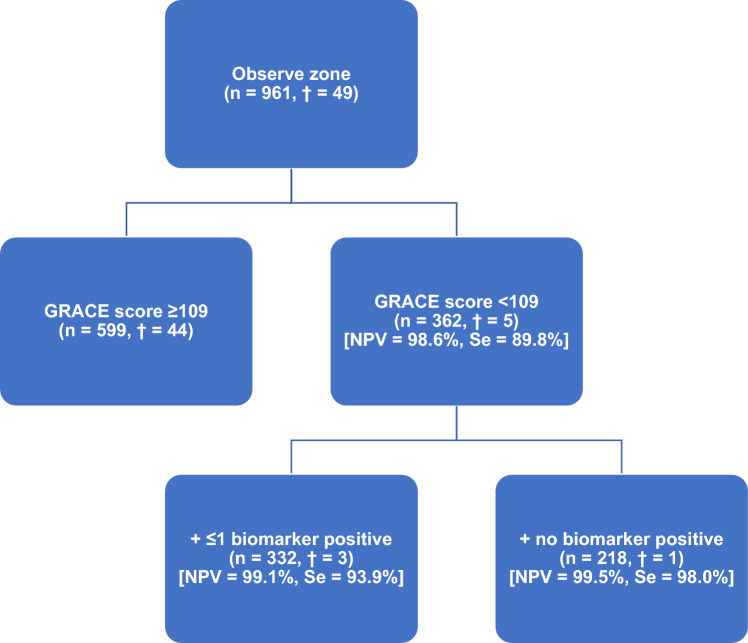
Classification and regression tree (CART) analysis of the RAPID-CPU cohort. NPV, negative predictive value; Se, sensitivity. **†**, represents the number of deaths within the cohort

**Fig. 3:**
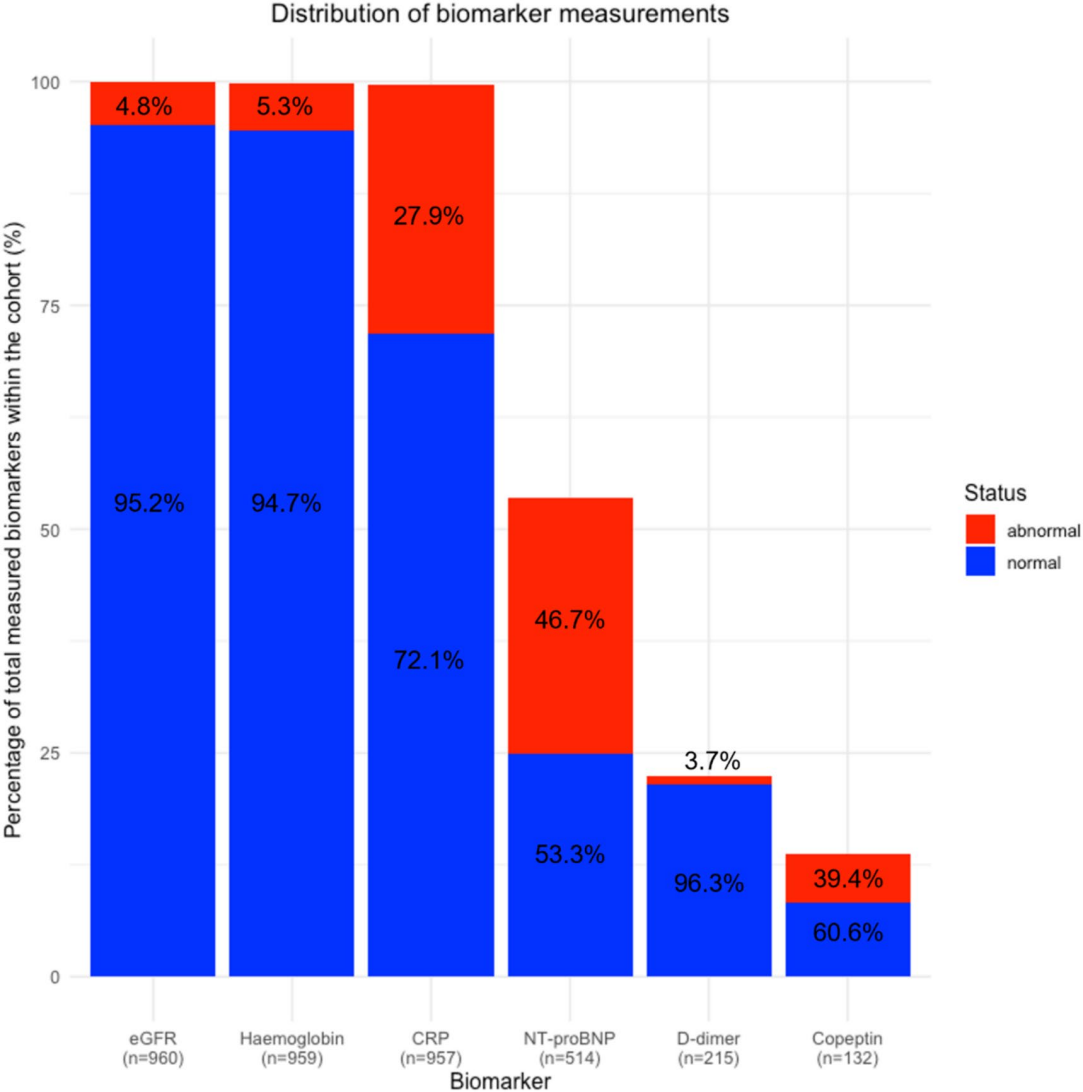
Frequency of biomarker measurements within the RAPID-CPU cohort. Histogram illustrating the distribution of biomarker measurements across the cohort. Biomarker names (eGFR, hemoglobin, CRP, NT-proBNP, D-dimer, and copeptin) are presented on the *x*-axis, while the *y*-axis denotes the percentage of measurements (%). Each bar is segmented into blue (indicating values within normal range) and red (indicating values above threshold), corresponding to normal and abnormal biomarker values, respectively. The percentages of normal and abnormal measurements are delineated within their respective segments. CRP, C-reactive protein; eGFR, estimated glomerular filtration rate; NT-proBNP, N-terminal pro-B-type natriuretic peptide

The prevalence of GRACE 1.0 score categories was 37.7% (*n* = 362) for low risk, 38% (*n* = 365) for intermediate risk, and 24.3% (*n* = 234) for high risk. Among patients with a low GRACE-score, 8.3% (*n* = 30) had > 1 biomarker elevation and were not considered for the present analysis. Figure 1 illustrates the mortality risk in relation to the GRACE-score, highlighting that a GRACE-score below 109 corresponds to a mortality risk below a 5% threshold. The CART analysis results are depicted in Fig. 2. Frequency of biomarker measurements for each candidate biomarker and the percentage of concentrations below cutoff are displayed in Fig. 3.

### GRACE-score < 109 and combined use of GRACE-score < 109 points together with biomarkers for outcome prediction: RAPID-CPU

**Fig. 4:**
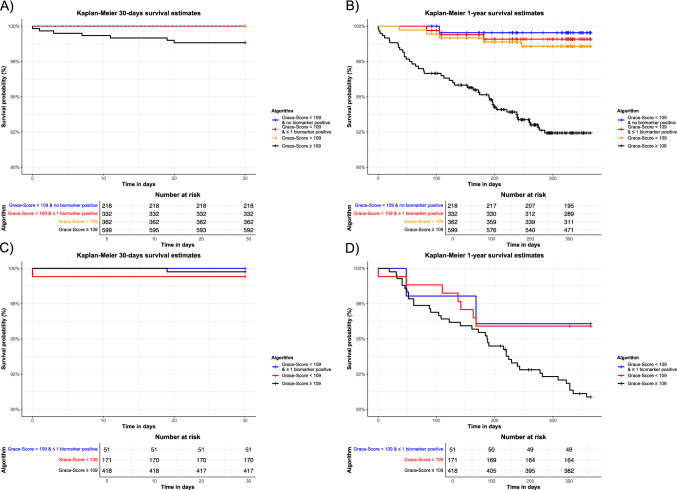
Kaplan–Meier survival curves illustrating the probability of survival within (**A**) 30 days and (**B**) 1 year for patients in the RAPID-CPU cohort, and within (**C**) 30 days and (**D**) 1 year for patients in the BACC cohort. BACC, Biomarkers in Acute Cardiac Care

**Fig. 5:**
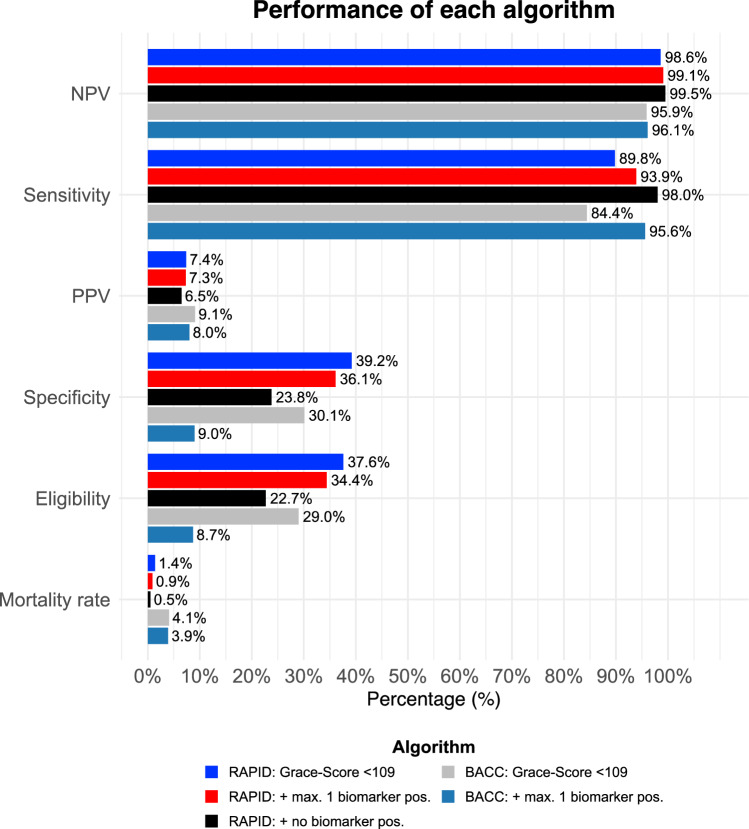
Comparison of each algorithm's performance for predicting all-cause mortality. BACC, Biomarkers in Acute Cardiac Care; NPV, negative predictive value; PPV, positive predictive value

Among patients with a GRACE-score < 109 points or ≤ 1 biomarkers above their respective cutoff, no deaths occurred within 30 days (Fig. 4A and supplement Fig. 2). Within a 365-day follow-up period, the mortality rate was 1.38% (5 out of 362) among those with a GRACE-score < 109 points, which decreased to 0.9% (3 out of 332) with ≤ 1 positive biomarker, and 0.46% (1 out of 218) in the absence of any elevated biomarker, corresponding to a 66.7% relative and a 0.92% absolute risk reduction. A GRACE 1.0 score < 109 points in the absence of any abnormal biomarker also reduced the false-negative rate for all-cause mortality from 10.2 to 2.0%. Figure 5 presents the performance metrics of this method, while Table 3 offers a detailed comparison. Regarding eligibility of the algorithm, restriction to patients with low GRACE 1.0 in combination with normal values in any of the tested biomarkers reduced the proportion of patients from 63 to 22.7% (218 of 961 patients) but achieved a high NPV of 99.5% and high sensitivity of 98%. Contrary, extension of eligibility to patients with a maximum of 1 abnormal biomarker increased the proportion of eligible patients from 22.7 to 34.5% (332 of 961 patients) without relevant decrease of NPV and sensitivity. Figure 4 presents Kaplan–Meier curves illustrating lower mortality rates with the combined usage of a low GRACE-score and at most one positive biomarker at 30 days (Fig. 4A) and at 1 year (Fig. 4B).

### Combined usage of GRACE-score and biomarkers for outcome prediction: BACC

The BACC cohort was used for external validation. Briefly, it consisted of a total of 2,303 patients, of whom 589 were classified into the observe zone. Overall, 1-year mortality rate was 7.6% (45 out of 589) during a median follow-up of 56 months. Baseline characteristics demonstrated similar trends and outcomes to our cohort. Non-survivors were older (median age of 82 years) and exhibited significantly higher GRACE 1.0 scores. Furthermore, cardiovascular (CV) history and CV risk factors were more prevalent, and concentrations of cardiac biomarkers were higher in non-survivors. Baseline characteristics of patients from the BACC cohort in the observe zone are shown in Table 2.

**Table 2:** Baseline characteristics of patients in the observe zone split by survival status, BACC cohort

	All (*n* = 589)	Non-survivor (*n* = 45)	Survivor (*n* = 544)	*p* value
Age [years], median (IQR)	74 (65–79)	82 (72.8–88)	74 (64–79)	0.0001
Female gender, *n* (%_all_)	170 (28.9)	17 (2.9)	153 (26.0)	< 0.0001
Heart rate [bpm], median (IQR)	79 (67–92)	84 (68–96.3)	79 (67–92)	0.9404
Systolic pressure [mmHg], median (IQR)	148 (130–165)	134 (116–160.5)	148 (130–165)	0.0077
GRACE-score, median (IQR)	123 (105–143)	150 (121.8–172.3)	122 (104–140)	< 0.0001
Low, * n* (%_all_)	171 (29)	7 (1.2)	164 (27.8)	0.0384
intermediate, * n* (%_all_)	260 (44.1)	9 (1.5)	251 (42.6)	0.0007
high, * n* (%_all_)	158 (26.8)	29 (4.9.2)	129 (21.9)	< 0.0001
*Symptoms*				
Time since onset < 3 h, * n* (%_all_)	151 (26.6)	13 (2.2)	138 (23.4)	0.0439
Chest pain, * n* (%_all_)	436 (74)	28 (4.8)	408 (69.3)	0.0605
*Laboratory*				
hs-cTnT 0 h [ng/L], median (IQR)	17 (13–25)	25 (17.8–35.3)	16 (13–24)	< 0.0001
hs-cTnT 1 h [ng/L], median (IQR)	16 (13–24)	23 (17–34.3)	16 (13–23)	0.0035
NT-proBNP [ng/L], median (IQR)	1194 (319–3679)	4352.5 (3124–10,304)	1050 (270.5–2764.5)	< 0.0001
GFR [mL/min/1.73 m^2]^], median (IQR)	62.2 (47.7–80.3)	49 (36.4–61.1)	64 (48.6–81.1)	< 0.0001
Copeptin [pmol/L], median (IQR)	9.7 (5.2–27.2)	20.8 (5.9–60.1)	9.1 (5.2–25.4)	0.0950
Hemoglobin [pg/L], median (IQR)	13.5 (12.4–14.6)	11.9 (10.9–12.8)	13.6 (12.5–14.7)	< 0.0001
CRP [mg/dL], median (IQR)	4.9 (4.9–10)	10 (4.9–28.3)	4.9 (4.9–9.0)	0.0013
*History*				
CAD, * n* (%_all_)	271 (46)	27 (4.6)	244 (41.4)	0.0503
Myocardial infarction, * n* (%_all_)	131 (22.2)	16 (2.7)	115 (19.5)	0.0256
Congestive Heart failure, * n* (%_all_)	113 (19.2)	13 (2.2)	100 (17)	0.0857
Smoking current, * n* (%_all_)	99 (38.8)	6 (1)	93 (15.8)	0.3152
Art. hypertension, * n* (%_all_)	474 (80.7)	38 (6.5)	436 (74)	0.5133
Diabetes mellitus, * n* (%_all_)	109 (18.7)	14 (2.4)	95 (16.1)	0.0268
Dyslipidemia, * n* (%_all_)	259 (44.0)	17 (2.9)	242 (41.1)	0.3840
Family history of CAD, * n* (%_all_)	68 (12.1)	1 (0.2)	67 (11.4)	0.0343
*Therapeutic work-up*				
Coronary angiography, * n* (%_all_)	165 (28.0)	7 (1.2)	158 (26.8)	0.0530
PCI, * n* (%_all_)	78 (13.2)	3 (0.5)	75 (12.7)	0.1760

bpm, beats per minute; CABG, coronary artery bypass graft; CAD, coronary artery disease; COPD, chronic obstructive pulmonary disease; CRP, C-reactive protein; GFR, glomerular filtration rate; Hs-cTnT, high-sensitivity cardiac troponin T; NT-proBNP, N-terminal pro-B-type natriuretic peptide; PCI, percutaneous coronary intervention. Percentages may not total 100 because of rounding

Upon validation in the BACC cohort, patients with a GRACE-score < 109 points and low biomarker-based risk showed a lower risk of adverse outcomes. Mortality rates decreased from 4.09% (7 out of 171) with a low GRACE 1.0 score to 3.92% (2 out of 51) when additionally ≤ 1 biomarkers were abnormal. No patients in the BACC cohort qualified for the lowest risk category, i.e., a low GRACE-score and normal biomarker panel. Accordingly, the proportion of patients qualifying for the new risk strategy was 8.7%. (51 of 589 patients). The NPV and sensitivity were 95.9% and 84.4% with a low GRACE-score, increasing to 96.1% and 95.6% when ≤ 1 biomarker was above specific cutoff, respectively (Fig. 5 and Table 3). Notably, within 30 days, one death occurred among patients with a GRACE-score < 109 points, but no deaths occurred when additionally ≤ 1 biomarker was above its respective cutoff. Figure 4 shows Kaplan–Meier curves illustrating lower mortality rates with the combined usage of a low GRACE-score and at most one positive biomarker at 30 days (Fig. 4C) and at 1 year (Fig. 4D).

**Table 3:** Comparison of algorithm performance metrics for predicting all-cause mortality

	NPV [%] [95% CI]	Se [%] [95% CI]	Sp [%] [95% CI]	PPV [%] [95% CI]	Eligibility [%]	FNR^a^ [%]
*RAPID-CPU*						
Grace-Score < 109	98.6 (96.9–99.4)	89.8 (77.8–96.6)	39.2 (36.0–42.4)	7.4 (6.7–8.1)	37.6	10.2
+ ≤ 1 biomarker positive	99.1 (97.3–99.7)	93.9 (83.1–98.7)	36.1 (33.0–39.3)	7.3 (6.8–7.9)	34.4	6.1
+ no biomarker positive	99.5 (96.9–99.9)	98.0 (89.2–99.9)	23.8 (21.1–26.7)	6.5 (6.1–6.8)	22.7	2.0
*BACC-Cohort*						
Grace-Score < 109	95.9 (92.1–97.9)	84.4 (70.6–93.5)	30.1 (26.3–34.2)	9.1 (8.0–10.3)	29	15.6
+ ≤ 1 biomarker positive	96.1 (86.0–99.0)	95.6 (84.9–99.5)	9.0 (6.7–11.7)	8.0 (7.5–8.5)	8.7	4.4

BACC, Biomarkers in Acute Cardiac Care; CPU, Chest pain unit; FNR, false-negative rate; NPV, negative predictive value; PPV, positive predictive value; Se, sensitivity; Sp, specificity

^a^The FNR represents the proportion of individuals who suffered death but were not detected by the algorithm

## Discussion

The generation of an observe zone is the consequence of fast triage protocols using hs-cTn [4, 5, 10, 24, 25]. While identification of patients at low and at high risk is optimized, this observe zone contains patients with miscellaneous differential diagnoses including acute and chronic cardiovascular diseases and non-ST-elevation myocardial infarction (NSTEMI) that escaped detection due to troponin rise beyond the recommended serial sampling interval of 1 h [4, 5, 25]. To reduce numbers of missed MI, 2020 ESC Guidelines and onwards [5], recommend a third troponin measurement at 3 h after admission. Furthermore, additional work-up emphasizing echocardiography is recommended to address the spectrum of potential differential diagnoses. The value of additional biomarkers that indicate pathomechanisms other than myocardial injury could help to diagnose differential diagnoses or relevant comorbidities among patients triaged as observe zone. However, measurement of additional biomarkers beyond hs-cTn and natriuretic peptides is currently not recommended by ESC Guidelines due to limited data on clinical consequences [25]. Focusing on prognostication rather than diagnosis, 2022 ACC Expert Consensus on Chest Pain in the Emergency Department [7] suggests implementing clinical risk scores. However, a recent secondary analysis of the HIGH-US study [8] revealed that risk scores are unlikely to improve triage without additional troponin measures and imaging. Given the paucity of evidence regarding the observe zone, our findings that address both diagnostic uncertainty and prognostication come timely. Our concept was to combine the prognostic information of the GRACE 1.0 score with the ability of certain biomarkers to rule out suspected diseases and associated risk. We included only biomarkers that were selected by attending physicians and used these biomarkers to rule out suspected acute heart failure, pulmonary embolism, or infection at pre-specified and guideline-recommended cutoffs [22, 26]. Simulating clinical routine in ACS management and similar to the strategy recommended to rule out pulmonary embolism, we used a CART analysis and first stratified patients by the GRACE 1.0 score and subsequently by the absence of abnormal biomarkers if the GRACE-score was < 109 points.

Our results are novel and contain four important findings:

First, the GRACE 1.0 score already enabled identification of a low-risk cohort with an annual mortality of < 5% compared to the overall 1-year mortality of 5.1% in the observe zone. This finding is not unexpected, since the GRACE 1.0 score contains prognostically relevant variables, including patient age, renal function, and information on congestive heart failure [9]. In clinical routine, the GRACE-score and other clinical scores are usually measured to confirm low risk or to determine the timing for invasive strategy in high-risk patients with confirmed ACS, at the moment. Evidence on the utility of the GRACE 1.0 score in other settings such as the observe zone is sparse and equivocal [8]. A noteworthy finding was the absence of mortality within the observe zone in patients with a GRACE-score < 109. Moreover, no patient died with a GRACE-score < 109 if ≤ 1 biomarker was above its respective cutoff. Our findings could help to expand the utility of the GRACE 1.0 score for risk assessment in the observe zone.

Second, addition of biomarker information to a low GRACE-score, i.e., the absence of abnormal concentrations of biomarkers that indicate infection, inflammation, chronic kidney disease (CKD), acute heart failure, pulmonary embolism, or anemia, improved both the clinical characterization of patients in the observe zone and concomitantly provide prognostic information. Our findings are heavily biased as the measurement of biomarkers was not obligatory and all additional biomarkers except hemoglobin, serum creatinine, and CRP were ordered at the discretion of the attending physician following a clinical suspicion. However, we do not perceive this bias as a limitation as biomarker measurements in the diagnostic process are part of the diagnostic work-up in real-world settings and are also recommended in guidelines on heart failure (natriuretic peptides) or pulmonary embolism (D-dimers, Copeptin) [22, 26].

Third, our strategy for risk stratification was validated in the BACC cohort, an observational study on 2,303 patients with suspected NSTE-ACS. In agreement with our results, a low GRACE-score in combination with a low biomarker-related risk as indicated by ≤ 1 abnormal biomarker was found to reduce mortality risk from 7.6 to 3.92%, with a still acceptable proportion of eligible patients of 20.3%. Sensitivity and NPV were 95.6% and 96.1% and thus slightly lower than in our derivation cohort. There are several reasons that may explain different performance. Overall mortality rate in the low GRACE-score category (1.38% vs 4.09%) is higher suggesting differences between the study populations regarding cardiovascular risk. These differences are further supported by the observation of higher rates of cardiovascular risk factors and comorbidities, a greater prevalence of the observe zone (14.2% vs. 25.6%), and disparities in the utilization of specific biomarkers, such as D-dimers (22.4% vs. 0%) and NT-pro BNP (53.5% vs. 37.7%). Although, our algorithm needs additional external validation in other observational studies, our study findings are promising. Notably, survivors exhibited a higher prevalence of certain cardiovascular comorbidities such as coronary artery disease, prior CABG, and dyslipidemia. While seemingly paradoxical, this observation likely reflects selection bias introduced by clinical triage decisions: non-survivors presented with significantly higher baseline GRACE risk scores and were therefore admitted more frequently for intensive diagnostic work-up and clinical care. This interpretation is further supported by the markedly lower discharge rate among patients who died within 1 year compared to survivors (22.4% vs. 49.8%, *p* = 0.0002). Thus, the higher prevalence of stable cardiovascular conditions among survivors represents appropriate clinical identification and outpatient management of relatively stable, known cardiovascular conditions, rather than indicating a protective effect of these comorbidities.

Fourth, until now traditional statistics using hs-cTn alone or in combination with clinical scores failed to improve risk stratification in the observe zone, probably owing to the complexity of underlying diseases. Our novel approach to test the discriminatory ability of a combination of the GRACE-score and miscellaneous representative biomarkers using CART analysis, a simple machine learning technique showed promising preliminary data. Seemingly, other biomarkers that reflect residual risk better and mirror other pathophysiological processes than the mild-to-moderate chronic myocardial injury that is characteristic for the observe zone enables a better discrimination of risk within the observe zone. It is tempting to speculate that more sophisticated ML-based algorithms than a CART analysis may further refine risk stratification in the observe zone.

## Limitations

Beyond hs-cTn, serum creatinine (and automatically calculated eGFR), hemoglobin, and CRP that were measured routinely in all patients, another three biomarkers (NT-pro BNP, D-dimers, and copeptin) could be requested by the treating physician depending on the clinical suspicion of underlying differential diagnoses. Accordingly, a full panel of additional biomarkers was not available for all patients and selection of biomarkers was systematically biased. However, our practice fully represents clinical reality where particular biomarkers, such as D-dimers and natriuretic peptides, are instrumented per guideline recommendations to facilitate diagnosis.

Our study findings are based on a single-center observational study with management of patients in a dedicated chest pain unit of an experienced tertiary referral center, led by a cardiologist and equipped with experienced and trained medical staff. Therefore, our findings cannot be generalized and should be validated in different clinical settings and geographical regions before broad implementation in clinical routine.

The need of accelerated triage and identification of low-risk patients is particularly important in busy emergency departments and limited ward capacities. While an MI may be missed in only a small fraction of patients in the observe zone, most deaths will occur after the initial 30 days after discharge. Although endpoints such as non-fatal MI would have strengthened clinical interpretation, we deliberately selected all-cause mortality as our primary endpoint, because it is an unequivocal and reliably adjudicated outcome. Given the observational nature of this study and practical challenges, structured follow-up and adjudication of MI or cardiovascular mortality would have been difficult due to subjective interpretation, and incomplete access to outpatient clinical data. Therefore, diagnostic work-up should ideally be completed as early as possible, either during index admission or early post-discharge. Albeit our findings on the ability of the proposed algorithm to identify patients at extremely low risk to die within 30 days, small numbers of events (1 death and no deaths respectively) is subject to sample size error and requires confirmation in larger populations.

## Conclusion

In conclusion, our study provides valuable insights into managing patients with suspected NSTE-ACS within the observe zone of the ESC 0/1-h algorithm. Combining the GRACE-score with an additional biomarker panel significantly reduced adverse outcomes and improved risk stratification and mortality prediction. This approach shows promise in identifying low-risk patients and optimizing management, particularly for those at acceptable mortality risk. Future research should explore additional biomarkers or employ machine learning algorithms to further refine risk prediction models for enhanced ACS management.

## Supplementary Information

Below is the link to the electronic supplementary material.

Supplementary file1 (DOCX 286 KB)

## Data Availability

The data underlying this article will be shared on reasonable request to the corresponding author.

## Abbreviations

ACC: American College of Cardiology
ACS: Acute Coronary Syndrome
AMI: Acute Myocardial Infarction
APACE: Advantageous Predictors of Acute Coronary Syndrome Evaluation
BACC: Biomarkers in Acute Cardiac Care
CA: Coronary Angiography
CAD: Coronary artery disease
CABG: Coronary artery bypass graft
ED: Emergency department
eGFR: Estimated Glomerular Filtration Rate
ESC: European Society of Cardiology
GRACE: Global Registry of Acute Coronary Events
MI: Myocardial Infarction
NSTEMI: Non-ST myocardial infarction
PCI: Percutaneous Coronary Intervention
STEMI: ST Elevation Myocardial Infarction
UAP: Unstable angina pectoris
