# Supplementary material for: Identification of patients with suspected NSTE-ACS in the observe zone: evaluating GRACE 1.0 score and a biomarker panel for risk stratification and management optimization
Source: Clin Res Cardiol. 2025 Apr 14;114(6):783–95. doi: 10.1007/s00392-025-02642-3 (PMC12089253; doi:10.1007/s00392-025-02642-3)
Supplement: Supplementary file 1 — Supplementary file1 (DOCX 286 KB) [file 392_2025_2642_MOESM1_ESM.docx]

**Supplemental material**

**Supplemental figures**

**Supplement figure 1. Flow chart of study recruitment and triage decision process.** This flow chart outlines the recruitment, exclusion, and triage decision processes for patients from 2016 to 2018 using ESC 0/1-hour hs-cTnT algorithm. Abbreviations: ACS, acute coronary syndrome; ESC, European Society of Cardiology; hs-cTnT, high-sensitivity cardiac troponin T; STEMI, ST‐segment–elevation myocardial infarction.

**Supplement figure 2.** **Time to event analysis for all-cause mortality within 30 days (A) and 365 days (B) in RAPID-CPU cohort.** Patients with a GRACE score <109 points or ≤1 positive biomarker demonstrated zero mortality within 30 days. This low-risk profile remained evident even after 1-year of follow-up.
